# 4-(Dimethyl­amino)pyridinium 4-toluene­sulfonate

**DOI:** 10.1107/S1600536808004856

**Published:** 2008-02-27

**Authors:** C. John McAdam, Jim Simpson

**Affiliations:** a Department of Chemistry, University of Otago, PO Box 56, Dunedin, New Zealand

## Abstract

In the title compound, C_7_H_11_N_2_^+^·C_7_H_7_O_3_S^−^, the cation is protonated at the N atom of the heterocyclic ring. The dimethyl­amino group lies close to the pyridinium ring plane with a dihedral angle between the pyridinium and the dimethyl­amine CNC planes of 3.82 (17)°. The N—C bond linking the dimethyl­amino substituent to the pyridinium ring is characteristically short [1.3360 (19) Å], suggesting some delocalization in the cation. In the crystal structure, N—H⋯O hydrogen bonds link individual pairs of cations and anions. The structure is further stabilized by an extensive series of C—H⋯O hydrogen bonds, augmented by π–π [centroid–centroid distance between adjacent pyridinium rings = 3.5807 (10) Å] and C—H⋯π inter­actions, giving a network structure.

## Related literature

For the preparation and uses of the title compound, see: Haynes & Indorato (1984 ▶); Moore, & Stupp (1990 ▶). For structures having the 4-(dimethyl­amino)pyridinium cation, see for example: Chao *et al.* (1977 ▶); Mayr-Stein & Bolte (2000 ▶); Sluka *et al.* (2003 ▶). For structures of salts of the 4-toluene­sulfonate anion with pyridinium or similar cations, see for example: Koshima *et al.* (2001 ▶, 2004 ▶); Biradha & Mahata (2005 ▶). For details of the Cambridge structural database, see: Allen (2002 ▶).
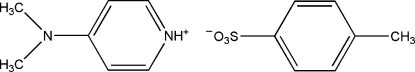


## Experimental

### 

#### Crystal data

C_7_H_11_N_2_^+^·C_7_H_7_O_3_S^−^
*M* *_r_* = 294.36
Monoclinic, 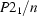

*a* = 8.9878 (7) Å
*b* = 17.5897 (12) Å
*c* = 9.8202 (6) Å
β = 111.429 (3)°
*V* = 1445.18 (17) Å^3^
*Z* = 4
Mo *K*α radiation
μ = 0.23 mm^−1^
*T* = 91 (2) K
0.43 × 0.07 × 0.04 mm

#### Data collection

Bruker APEXII CCD area-detector diffractometer
Absorption correction: multi-scan (*SADABS*; Bruker, 2006 ▶) *T*_min_ = 0.860, *T*_max_ = 0.991
22414 measured reflections
3792 independent reflections
3087 reflections with *I* > 2σ(*I*)
*R*_int_ = 0.037

#### Refinement

*R*[*F* ^2^ > 2σ(*F* ^2^)] = 0.042
*wR*(*F* ^2^) = 0.125
*S* = 1.05
3792 reflections
188 parameters
H atoms treated by a mixture of independent and constrained refinement
Δρ_max_ = 1.11 e Å^−3^
Δρ_min_ = −0.41 e Å^−3^

### 

Data collection: *APEX2* (Bruker, 2006 ▶); cell refinement: *APEX2* and *SAINT* (Bruker, 2006 ▶); data reduction: *SAINT*; program(s) used to solve structure: *SHELXS97* (Sheldrick, 2008 ▶) and *TITAN2000* (Hunter & Simpson, 1999 ▶); program(s) used to refine structure: *SHELXL97* (Sheldrick, 2008 ▶) and *TITAN2000*; molecular graphics: *ORTEP-3* (Farrugia, 1997 ▶) and *Mercury* (Macrae *et al.*, 2006 ▶); software used to prepare material for publication: *SHELXL97*, *enCIFer* (Allen *et al.*, 2004 ▶) and *PLATON* (Spek, 2003 ▶).

## Supplementary Material

Crystal structure: contains datablocks global, I. DOI: 10.1107/S1600536808004856/ng2425sup1.cif

Structure factors: contains datablocks I. DOI: 10.1107/S1600536808004856/ng2425Isup2.hkl

Additional supplementary materials:  crystallographic information; 3D view; checkCIF report

## Figures and Tables

**Table 1:** Hydrogen-bond geometry (Å, °)

*D*—H⋯*A*	*D*—H	H⋯*A*	*D*⋯*A*	*D*—H⋯*A*
C8—H8⋯O3	0.95	2.40	3.201 (2)	142
N1—H1⋯O3^i^	0.81 (2)	1.92 (2)	2.7160 (18)	171 (2)
C12—H12⋯O2^i^	0.95	2.64	3.376 (2)	135
C7—H7*A*⋯O2^ii^	0.98	2.62	3.553 (3)	160
C13—H13*C*⋯O1^ii^	0.98	2.56	3.408 (2)	145
C6—H6⋯O1^iii^	0.95	2.63	3.490 (2)	151
C9—H9⋯O1^iii^	0.95	2.44	3.350 (2)	160
C13—H13*A*⋯O2^iii^	0.98	2.56	3.502 (2)	161
C14—H14*A*⋯O3^iv^	0.98	2.67	3.541 (2)	148
C11—H11⋯*Cg*2^v^	0.95	2.72	3.5883 (18)	152

Symmetry codes: (i) 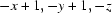; (ii) 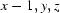; (iii) 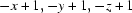; (iv) 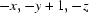; (v) 
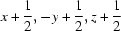
. *Cg*2 is the centroids of the C1–C6 ring.

**Fig. 1.**
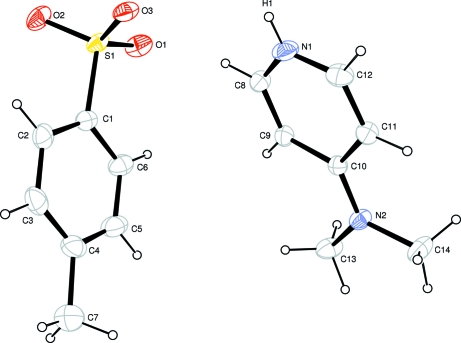
The asymmetric unit of (I), with atom labels and 50% probability displacement ellipsoids for non-H atoms.

**Fig. 2.**
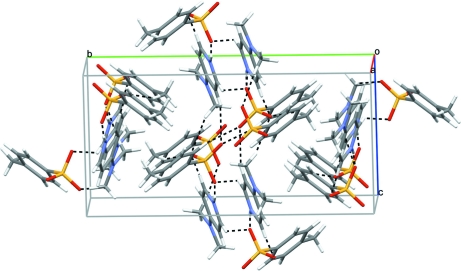
Crystal packing of (I) with hydrogen bonds drawn as dashed lines.

**Fig. 3.**
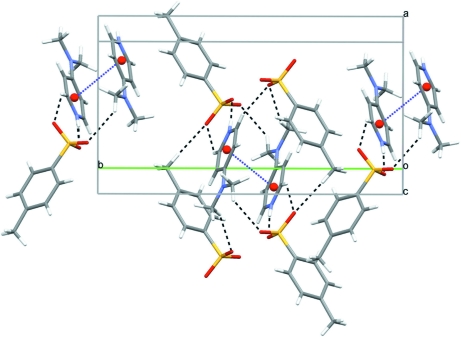
π···π stacking (dotted lines) between adjacent pyridinium rings of (I). The red circles represent pyridinium ring centroids separated by 3.5807 (10) Å. Additional hydrogen bonding interactions are shown as dashed lines.

## supplementary crystallographic information

### Comment

The title compound (I) was first reported and characterized as a side product by Haynes and Indorato (1984). However, it is better known under the acronym DPTS following the work of Moore and Stupp (1990) for its role as a convenient provider of stoichiometric quantities of anhydrous *p*-toluenesulfonic acid (PTSA) and 4-(dimethylamino)pyridine (DMAP) for the catalytic synthesis of polyesters at room temperature. Our interest in the synthesis of organometallic polyesters required the synthesis of DPTS and its structure is reported here, Fig 1.

The asymmetric unit of (I), C_7_H_11_N_2_^+^, C_7_H_7_O_3_S^-^, consists of a 4-(dimethylamino)pyridinium cation and a 4-toluenesulfonate anion. In common with other DMAPH^+^ cations (Chao *et al.*, 1977; Mayr-Stein & Bolte, 2000; Sluka *et al.*, 2003), protonation is at the N1 atom of the pyridinium ring. Bond distances and angles in both the cation and anion are normal (Allen *et al.*, 1987) and those in the anion are comparable to those in other 4-toluenesulfonate salts (Koshima *et al.*, 2001, 2004; Biradha & Mahata 2005). The N2—C10 bond linking the dimethylamino substituent to the pyridinium ring is short, 1.3360 (19)Å suggesting some delocalization in the cation. The fact that the dimethylamino group lies close to the plane of the pyridinium ring, with a dihedral between the pyridinium and the dimethylamine C13N2C14 planes of 3.82 (17)°, supports this observation as does the fact that the C10N2C13C14 system is reasonably planar with an r.m.s. deviation of 0.006 Å. A search of the Cambridge structural database (Allen, 2002) reveals 47 similar structures incorporating the 4-(dimethylamino)pyridinium cation for which the mean corresponding N—C distance is 1.34 (1) Å.

In the crystal structure N—H···O hydrogen bonds link individual pairs of cations and anions and the structure is further stabilized by an extensive network of C—H···O hydrogen bonds, Fig. 2, Table 1. In addition π···π stacking beween adjacent pyridinium rings (*Cg*1···*Cg*1 = 3.5807 (10) Å), Fig. 3, and C11—H11···*Cg*2 interactions also contribute to the crystal packing. (*Cg*1 & *Cg*2 are the centroids of the N1, C8···C12 and C1···C6 rings respectively).

### Experimental

The title compound was prepared according to the method of Moore and Stupp (1990) with X-ray quality crystals grown from 1,2-dichloroethane.

### Refinement

The H1 atom involved in N—H···O hydrogen bonding was located in a difference Fourier map and was freely refined with an isotropic displacement parameter. All H-atoms bound to carbon were refined using a riding model with d(C—H) = 0.95 Å, *U*_iso_=1.2*U*_eq_ (C) for aromatic and 0.98 Å, *U*_iso_ = 1.5*U*_eq_ (C) for CH_3_ H atoms. The highest residual electron density peak is located at 0.76 Å from H2.

### Crystal data

**Table:**

C_7_H_11_N_2_^+^·C_7_H_7_O_3_S^–^	*F*_000_ = 624
*M**_r_* = 294.36	*D*_x_ = 1.353 Mg m^−^^3^
Monoclinic, *P*2_1_/*n*	Mo *K*α radiation λ = 0.71073 Å
Hall symbol: -P 2yn	Cell parameters from 5199 reflections
*a* = 8.9878 (7) Å	θ = 2.3–28.4º
*b* = 17.5897 (12) Å	µ = 0.23 mm^−^^1^
*c* = 9.8202 (6) Å	*T* = 91 (2) K
β = 111.429 (3)º	Block, colourless
*V* = 1445.18 (17) Å^3^	0.43 × 0.07 × 0.04 mm
*Z* = 4	

### Data collection

**Table:**

Bruker APEXII CCD area-detector diffractometer	3792 independent reflections
Radiation source: fine-focus sealed tube	3087 reflections with *I* > 2σ(*I*)
Monochromator: graphite	*R*_int_ = 0.037
*T* = 91(2) K	θ_max_ = 28.9º
ω scans	θ_min_ = 2.7º
Absorption correction: multi-scan(SADABS; Bruker, 2006)	*h* = −12→11
*T*_min_ = 0.860, *T*_max_ = 0.991	*k* = −23→23
22414 measured reflections	*l* = −13→13

### Refinement

**Table:**

Refinement on *F*^2^	Secondary atom site location: difference Fourier map
Least-squares matrix: full	Hydrogen site location: inferred from neighbouring sites
*R*[*F*^2^ > 2σ(*F*^2^)] = 0.042	H atoms treated by a mixture of independent and constrained refinement
*wR*(*F*^2^) = 0.125	*w* = 1/[σ^2^(*F*_o_^2^) + (0.0662*P*)^2^ + 0.7048*P*] where *P* = (*F*_o_^2^ + 2*F*_c_^2^)/3
*S* = 1.05	(Δ/σ)_max_ = 0.001
3792 reflections	Δρ_max_ = 1.11 e Å^−^^3^
188 parameters	Δρ_min_ = −0.40 e Å^−^^3^
Primary atom site location: structure-invariant direct methods	Extinction correction: none

### Special details

**Table:**

Experimental. As the crystals were weakly diffracting data was collected using 55 sec exposures per frame.
Geometry. All e.s.d.'s (except the e.s.d. in the dihedral angle between two l.s. planes) are estimated using the full covariance matrix. The cell e.s.d.'s are taken into account individually in the estimation of e.s.d.'s in distances, angles and torsion angles; correlations between e.s.d.'s in cell parameters are only used when they are defined by crystal symmetry. An approximate (isotropic) treatment of cell e.s.d.'s is used for estimating e.s.d.'s involving l.s. planes.
Refinement. Refinement of *F*^2^ against ALL reflections. The weighted *R*-factor *wR* and goodness of fit *S* are based on *F*^2^, conventional *R*-factors *R* are based on *F*, with *F* set to zero for negative *F*^2^. The threshold expression of *F*^2^ > σ(*F*^2^) is used only for calculating *R*-factors(gt) *etc*. and is not relevant to the choice of reflections for refinement. *R*-factors based on *F*^2^ are statistically about twice as large as those based on *F*, and *R*- factors based on ALL data will be even larger.

### Fractional atomic coordinates and isotropic or equivalent isotropic displacement parameters (Å^2^)

**Table:**

	*x*	*y*	*z*	*U*_iso_*/*U*_eq_	
S1	0.62942 (4)	0.40655 (2)	0.30685 (4)	0.02071 (12)	
O1	0.65251 (15)	0.46441 (8)	0.41760 (13)	0.0300 (3)	
O2	0.76431 (14)	0.35591 (8)	0.33293 (14)	0.0305 (3)	
O3	0.57456 (14)	0.43952 (7)	0.15893 (12)	0.0253 (3)	
C1	0.46612 (19)	0.35035 (9)	0.30817 (17)	0.0218 (3)	
C2	0.4183 (2)	0.28743 (10)	0.21659 (18)	0.0262 (3)	
H2	0.4773	0.2724	0.1582	0.031*	
C3	0.2834 (2)	0.24663 (10)	0.2110 (2)	0.0320 (4)	
H3	0.2503	0.2040	0.1477	0.038*	
C4	0.1961 (2)	0.26748 (11)	0.2972 (2)	0.0338 (4)	
C5	0.2470 (2)	0.32962 (12)	0.3887 (2)	0.0336 (4)	
H5	0.1893	0.3440	0.4487	0.040*	
C6	0.3805 (2)	0.37166 (11)	0.39512 (18)	0.0275 (4)	
H6	0.4129	0.4145	0.4581	0.033*	
C7	0.0465 (3)	0.22552 (14)	0.2912 (3)	0.0531 (6)	
H7A	−0.0405	0.2620	0.2757	0.080*	
H7B	0.0674	0.1985	0.3835	0.080*	
H7C	0.0160	0.1889	0.2103	0.080*	
N1	0.24038 (18)	0.56935 (9)	0.00587 (17)	0.0267 (3)	
H1	0.301 (3)	0.5637 (13)	−0.037 (2)	0.036 (6)*	
C8	0.2816 (2)	0.54668 (9)	0.14560 (19)	0.0250 (3)	
H8	0.3848	0.5256	0.1948	0.030*	
C9	0.17879 (18)	0.55323 (9)	0.21827 (17)	0.0207 (3)	
H9	0.2105	0.5373	0.3173	0.025*	
C10	0.02343 (18)	0.58410 (8)	0.14474 (16)	0.0172 (3)	
C11	−0.0123 (2)	0.60940 (9)	−0.00116 (17)	0.0211 (3)	
H11	−0.1129	0.6322	−0.0537	0.025*	
C12	0.0971 (2)	0.60106 (10)	−0.06569 (18)	0.0254 (3)	
H12	0.0717	0.6180	−0.1634	0.031*	
N2	−0.08300 (16)	0.58856 (8)	0.21026 (14)	0.0206 (3)	
C13	−0.0458 (2)	0.55874 (11)	0.35772 (17)	0.0280 (4)	
H13A	0.0303	0.5927	0.4286	0.042*	
H13B	0.0013	0.5080	0.3647	0.042*	
H13C	−0.1441	0.5556	0.3789	0.042*	
C14	−0.2376 (2)	0.62548 (12)	0.13823 (19)	0.0296 (4)	
H14A	−0.2949	0.6003	0.0447	0.044*	
H14B	−0.2210	0.6791	0.1208	0.044*	
H14C	−0.3008	0.6218	0.2009	0.044*	

### Atomic displacement parameters (Å^2^)

**Table:**

	*U*^11^	*U*^22^	*U*^33^	*U*^12^	*U*^13^	*U*^23^
S1	0.01460 (19)	0.0296 (2)	0.01777 (19)	0.00256 (14)	0.00579 (14)	0.00122 (14)
O1	0.0244 (6)	0.0395 (7)	0.0268 (6)	−0.0059 (5)	0.0103 (5)	−0.0087 (5)
O2	0.0181 (6)	0.0409 (7)	0.0314 (6)	0.0095 (5)	0.0077 (5)	0.0044 (5)
O3	0.0191 (6)	0.0375 (7)	0.0220 (6)	0.0038 (5)	0.0107 (4)	0.0071 (5)
C1	0.0185 (7)	0.0270 (8)	0.0187 (7)	0.0030 (6)	0.0052 (6)	0.0067 (6)
C2	0.0254 (8)	0.0247 (8)	0.0262 (8)	0.0076 (7)	0.0069 (6)	0.0069 (6)
C3	0.0305 (9)	0.0217 (8)	0.0368 (10)	0.0022 (7)	0.0040 (8)	0.0065 (7)
C4	0.0253 (9)	0.0304 (9)	0.0448 (11)	0.0019 (7)	0.0118 (8)	0.0155 (8)
C5	0.0282 (9)	0.0409 (11)	0.0368 (10)	0.0026 (8)	0.0181 (8)	0.0090 (8)
C6	0.0244 (8)	0.0359 (10)	0.0235 (8)	0.0010 (7)	0.0102 (7)	0.0030 (7)
C7	0.0366 (12)	0.0412 (12)	0.0846 (19)	0.0004 (9)	0.0258 (12)	0.0119 (12)
N1	0.0268 (7)	0.0282 (8)	0.0331 (8)	−0.0029 (6)	0.0205 (6)	−0.0057 (6)
C8	0.0209 (8)	0.0213 (8)	0.0347 (9)	0.0010 (6)	0.0126 (7)	−0.0004 (7)
C9	0.0189 (7)	0.0191 (7)	0.0229 (7)	0.0016 (6)	0.0064 (6)	0.0018 (6)
C10	0.0183 (7)	0.0161 (7)	0.0172 (7)	−0.0013 (5)	0.0067 (5)	−0.0020 (5)
C11	0.0224 (8)	0.0230 (8)	0.0176 (7)	0.0009 (6)	0.0070 (6)	0.0008 (6)
C12	0.0295 (9)	0.0285 (9)	0.0208 (7)	−0.0042 (7)	0.0121 (6)	−0.0021 (6)
N2	0.0173 (6)	0.0286 (7)	0.0164 (6)	0.0026 (5)	0.0067 (5)	0.0020 (5)
C13	0.0232 (8)	0.0437 (10)	0.0184 (7)	0.0004 (7)	0.0093 (6)	0.0057 (7)
C14	0.0185 (8)	0.0445 (10)	0.0259 (8)	0.0094 (7)	0.0082 (6)	0.0047 (7)

### Geometric parameters (Å, °)

**Table:**

S1—O1	1.4481 (13)	N1—C8	1.344 (2)
S1—O2	1.4499 (12)	N1—H1	0.81 (2)
S1—O3	1.4718 (11)	C8—C9	1.363 (2)
S1—C1	1.7735 (17)	C8—H8	0.9500
C1—C2	1.391 (2)	C9—C10	1.425 (2)
C1—C6	1.394 (2)	C9—H9	0.9500
C2—C3	1.392 (3)	C10—N2	1.3360 (19)
C2—H2	0.9500	C10—C11	1.420 (2)
C3—C4	1.397 (3)	C11—C12	1.359 (2)
C3—H3	0.9500	C11—H11	0.9500
C4—C5	1.382 (3)	C12—H12	0.9500
C4—C7	1.515 (3)	N2—C13	1.4595 (19)
C5—C6	1.391 (3)	N2—C14	1.461 (2)
C5—H5	0.9500	C13—H13A	0.9800
C6—H6	0.9500	C13—H13B	0.9800
C7—H7A	0.9800	C13—H13C	0.9800
C7—H7B	0.9800	C14—H14A	0.9800
C7—H7C	0.9800	C14—H14B	0.9800
N1—C12	1.342 (2)	C14—H14C	0.9800
			
O1—S1—O2	114.71 (8)	C8—N1—H1	120.4 (16)
O1—S1—O3	111.64 (8)	N1—C8—C9	121.48 (16)
O2—S1—O3	111.92 (7)	N1—C8—H8	119.3
O1—S1—C1	106.15 (8)	C9—C8—H8	119.3
O2—S1—C1	107.29 (8)	C8—C9—C10	119.56 (15)
O3—S1—C1	104.31 (7)	C8—C9—H9	120.2
C2—C1—C6	120.03 (16)	C10—C9—H9	120.2
C2—C1—S1	119.99 (13)	N2—C10—C11	121.97 (14)
C6—C1—S1	119.89 (14)	N2—C10—C9	121.33 (14)
C1—C2—C3	119.64 (16)	C11—C10—C9	116.70 (14)
C1—C2—H2	120.2	C12—C11—C10	120.08 (15)
C3—C2—H2	120.2	C12—C11—H11	120.0
C2—C3—C4	120.97 (18)	C10—C11—H11	120.0
C2—C3—H3	119.5	N1—C12—C11	121.34 (15)
C4—C3—H3	119.5	N1—C12—H12	119.3
C5—C4—C3	118.39 (17)	C11—C12—H12	119.3
C5—C4—C7	119.3 (2)	C10—N2—C13	120.79 (13)
C3—C4—C7	122.3 (2)	C10—N2—C14	120.98 (13)
C4—C5—C6	121.65 (17)	C13—N2—C14	118.20 (13)
C4—C5—H5	119.2	N2—C13—H13A	109.5
C6—C5—H5	119.2	N2—C13—H13B	109.5
C5—C6—C1	119.31 (17)	H13A—C13—H13B	109.5
C5—C6—H6	120.3	N2—C13—H13C	109.5
C1—C6—H6	120.3	H13A—C13—H13C	109.5
C4—C7—H7A	109.5	H13B—C13—H13C	109.5
C4—C7—H7B	109.5	N2—C14—H14A	109.5
H7A—C7—H7B	109.5	N2—C14—H14B	109.5
C4—C7—H7C	109.5	H14A—C14—H14B	109.5
H7A—C7—H7C	109.5	N2—C14—H14C	109.5
H7B—C7—H7C	109.5	H14A—C14—H14C	109.5
C12—N1—C8	120.78 (14)	H14B—C14—H14C	109.5
C12—N1—H1	118.9 (16)		
			
O1—S1—C1—C2	−177.44 (13)	C2—C1—C6—C5	−0.3 (2)
O2—S1—C1—C2	−54.35 (14)	S1—C1—C6—C5	176.27 (13)
O3—S1—C1—C2	64.53 (14)	C12—N1—C8—C9	1.7 (3)
O1—S1—C1—C6	6.04 (15)	N1—C8—C9—C10	0.6 (2)
O2—S1—C1—C6	129.12 (14)	C8—C9—C10—N2	177.23 (15)
O3—S1—C1—C6	−112.00 (14)	C8—C9—C10—C11	−2.5 (2)
C6—C1—C2—C3	0.8 (2)	N2—C10—C11—C12	−177.39 (15)
S1—C1—C2—C3	−175.68 (12)	C9—C10—C11—C12	2.3 (2)
C1—C2—C3—C4	−0.6 (3)	C8—N1—C12—C11	−1.8 (3)
C2—C3—C4—C5	−0.2 (3)	C10—C11—C12—N1	−0.2 (3)
C2—C3—C4—C7	178.49 (18)	C11—C10—N2—C13	176.99 (15)
C3—C4—C5—C6	0.8 (3)	C9—C10—N2—C13	−2.7 (2)
C7—C4—C5—C6	−177.93 (19)	C11—C10—N2—C14	−4.9 (2)
C4—C5—C6—C1	−0.6 (3)	C9—C10—N2—C14	175.34 (15)

### Hydrogen-bond geometry (Å, °)

**Table:**

*D*—H···*A*	*D*—H	H···*A*	*D*···*A*	*D*—H···*A*
C8—H8···O3	0.95	2.40	3.201 (2)	142
N1—H1···O3^i^	0.81 (2)	1.92 (2)	2.7160 (18)	171 (2)
C12—H12···O2^i^	0.95	2.64	3.376 (2)	135
C7—H7A···O2^ii^	0.98	2.62	3.553 (3)	160
C13—H13C···O1^ii^	0.98	2.56	3.408 (2)	145
C6—H6···O1^iii^	0.95	2.63	3.490 (2)	151
C9—H9···O1^iii^	0.95	2.44	3.350 (2)	160
C13—H13A···O2^iii^	0.98	2.56	3.502 (2)	161
C14—H14A···O3^iv^	0.98	2.67	3.541 (2)	148
C11—H11···Cg2^v^	0.95	2.72	3.5883 (18)	152

Symmetry codes: (i) −*x*+1, −*y*+1, −*z*; (ii) *x*−1, *y*, *z*; (iii) −*x*+1, −*y*+1, −*z*+1; (iv) −*x*, −*y*+1, −*z*; (v) *x*+1/2, −*y*+1/2, *z*+1/2.
